# Progress on the Synthesis and Application of CuSCN Inorganic Hole Transport Material in Perovskite Solar Cells

**DOI:** 10.3390/ma11122592

**Published:** 2018-12-19

**Authors:** Funeka Matebese, Raymond Taziwa, Dorcas Mutukwa

**Affiliations:** 1 Fort Hare Institute of Technology, University of Fort Hare, Alice 5700, South Africa; fmatebese@ufh.ac.za; 2 Department of Chemistry, University of Fort Hare, Alice 5700, South Africa; dmutukwa@ufh.ac.za

**Keywords:** perovskite solar cells, hole transport materials, inorganic hole transport materials, CuSCN

## Abstract

*P*-type wide bandgap semiconductor materials such as CuI, NiO, Cu_2_O and CuSCN are currently undergoing intense research as viable alternative hole transport materials (HTMs) to the spiro-OMeTAD in perovskite solar cells (PSCs). Despite 23.3% efficiency of PSCs, there are still a number of issues in addition to the toxicology of Pb such as instability and high-cost of the current HTM that needs to be urgently addressed. To that end, copper thiocyanate (CuSCN) HTMs in addition to robustness have high stability, high hole mobility, and suitable energy levels as compared to spiro-OMeTAD HTM. CuSCN HTM layer use affordable materials, require short synthesis routes, require simple synthetic techniques such as spin-coating and doctor-blading, thus offer a viable way of developing cost-effective PSCs. HTMs play a vital role in PSCs as they can enhance the performance of a device by reducing charge recombination processes. In this review paper, we report on the current progress of CuSCN HTMs that have been reported to date in PSCs. CuSCN HTMs have shown enhanced stability when exposed to weather elements as the solar devices retained their initial efficiency by a greater percentage. The efficiency reported to date is greater than 20% and has a potential of increasing, as well as maintaining thermal stability.

## 1. Introduction

We have recently witnessed the rise of perovskite solar cells (PSCs) based on organic–inorganic halide perovskites as the next generation of thin-film solar cells. They have managed to rise above their predecessors, dye-sensitized solar cells (DSSCs) from an efficiency of 3.8% to 23.3% in less than 10 years of research [1,2,3]. The keen interest in PSCs and their exceptional performance can be attributed to the excellent properties of the perovskite light absorbers which include long diffusion lengths, defect tolerance, strong absorption coefficient, low recombination rates, ease of fabrication and high charge mobility as well as favorable bandgaps [4,5,6]. However, commercialization of PSCs has been limited due to stability, carrier lifetime and current-voltage hysteresis issues [7]. Perovskite materials are known to degrade when exposed to moisture, heat, oxygen and UV radiation. Consequently, research efforts are now focused on improving the stability and cell performance issues of PSCs to pave way for commercialization. 

**Figure 1:**
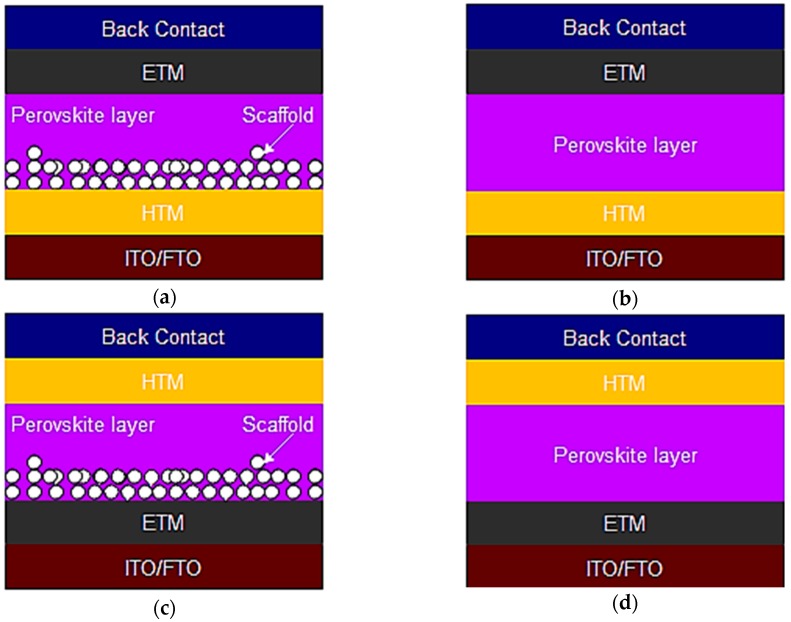
Different PSC device architecture (**a**) Inverted mesoscopic (p-i-n) device (**b**) Inverted planar (p-i-n) device (**c**) Mesoscopic (n-i-p) device (**d**) Planar (n-i-p) device.

The PSC is made up of an active perovskite layer, an HTM, an electron transport material (ETM), a back electrode, a transparent electrode and or a mesoporous TiO_2_ layer. Figure 1 presents the schematic diagrams of the mesoscopic and planar PSC device architecture.

The first solid-state PSC was introduced by Kim et al. [8]. The PSC consisted of methylammonium lead iodide (MAPbI_3_) as a sensitizer, with a spiro-OMeTAD (2,2’,7,7’-tetra-kis (*N*,*N*-di-*p*-methoxyphenylamine)-9,9’-spirobifluorene) as an HTM and nanoporous TiO_2_ as an ETM. This device architecture had an efficiency of 9.7% and was similar to that of DSSCs, with the liquid electrolyte replaced by the solid spiro-OMeTAD. Introduction of a solid-state HTM led to a significant increase in the efficiency of PSCs. This might have been due to better stability as compared to the first PSC which was introduced by Miyasaka and co-workers [9]. In their work, they employed a liquid electrolyte tri-iodide redox couple (I_3_^−^/I^−^) employed in DSSCs and achieved an efficiency of 3.8% [9]. The low efficiency was as a result of the perovskite material dissolving in the tri-iodide redox couple electrolyte. Even though the use of spiro-OMeTAD led to improved performance, spiro-OMeTAD suffers from low hole mobility of about 6 × 10^−5^ cm^2^ V^−1^ s^−1^ and low electrical conductivity in its pristine state. In order to improve the performance of spiro-OMeTAD, additives such as Li-TFSI lithium bis (trifluoromethanesulfonyl)imide and silver bis (trifluoromethanesulfonyl)imide (AgTFSI) have been commonly adopted [10,11].

However, the use of these additives resulted in the degradation of the perovskite layer, thus leading to long-term PSCs instability. Moreover, use of spiro-OMeTAD as HTM in PSCs present several limitations as it is costly to manufacture and the synthesis routes are tedious which does not augur well for large-scale fabrication, thus making it not viable for commercial production [10,11]. Therefore, research efforts have been dedicated to finding alternative HTMs of improved stability and hole mobility as they have a significant contribution to the performance of PSCs [12]. Since the introduction of spiro-OMeTAD, various organic HTMs such as PEDOT:PSS (poly(3,4-ethylenedioxythiophene)-poly (styrenesulfonate)), PTAA (poly(triarylamine)), triphenylamine-based molecules (TPA), carbazole-based molecules, spiro-OMeTAD derivatives and thiophene-based molecules have been tested in PSCs [13,14,15]. All these research efforts were centered at finding affordable HTMs with improved hole mobility, stability, and a feasible synthesis route. 

In addition to organic HTMs, inorganic HTMs such as CuI, CuSCN, NiO, and Cu_2_O which potentially have better stability than organic HTMs have also been tested in PSCs [16,17,18]. Christians and co-workers [19] were the first to apply inorganic HTM materials in PSCs. They fabricated copper iodide (CuI) on regular-structured PSC and obtained a conversion efficiency of 6% with low open-circuit voltage (V_OC_) of 0.79 V that was due to high recombination levels. The PCE of the CuI-based HTM swiftly reached 16.8% in inverted planar architecture [20]. Inherent advantages, such as the ability to reduce the production costs, suitable energy levels, high hole mobility as well as enhancement of its resistance to degradation, make inorganic HTMs a promising class of materials to replace spiro-OMeTAD [21,22]. In addition, the stability of PSCs has been improved by inorganic HTMs and the conversion efficiency has rapidly increased in the past few years [23]. However, it has been reported that most solvents used for inorganic material deposition dissolve the perovskite materials, and this could be the reason why there is a dearth of scientific reports that report on applications of inorganic HTMs in PSCs. One such promising inorganic HTM is CuSCN, which is an inexpensive and abundant metal pseudohalide of singly ionized copper. It has a well-aligned work function and has demonstrated high mobility as well as good thermal stability [24]. Therefore, this review is focused on reporting the progress of CuSCN inorganic HTMs. This review also discusses the various fabrication routes employed up to date to prepare CuSCN-based PSCs. This review paper also evaluates the photovoltaic performance of the tested solar cell devices employing CuSCN HTMs. Lastly, the review paper presents the various architectures employed in the PSCs. The triumphs and challenges of the CuSCN HTMs in PSC are presented here in detail. 

## 2. Roles and Ideal Characteristics of HTM 

Hole transport materials play different crucial roles, such as: (i) blocking electron transfer to the back-contact metal electrode, (ii) extraction of photo-generated holes from the perovskite and transportation of these extracted charges back to the back-contact metal electrode, and (iii) prevent direct contact between the metal electrode and the perovskite layer [25,26,27,28,29]. An ideal HTM should exhibit properties, such as: (i) high hole mobility, chemical stability and thermal robustness to withstand annealing process during fabrication of the PSC; (ii) the highest occupied molecular orbital (HOMO) energy level should match the valence band energy of the perovskite; (iii) should protect the perovskite layer from air and moisture and be able to prevent the diffusion of external moieties or elements inside the photo-absorber. Moreover, the HTM should have a low annealing temperature and short annealing times to avoid degradation of the underlying layers. Last but not least, the HTM (iv) should show minimum absorption in the visible and near-infrared of the solar spectrum to avoid absorption of photons during the photo-excitation of excitons [29,30,31,32,33].

**Figure 2:**
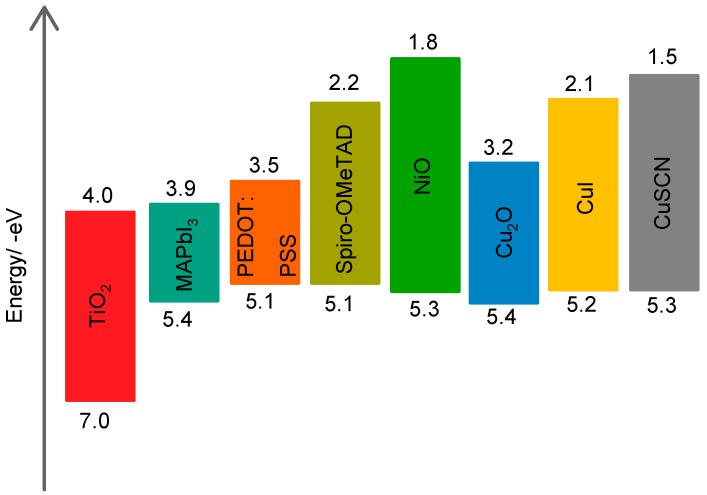
Schematic of the energy levels of CuSCN, perovskite material, TiO_2_ ETM and various HTMs [34,35].

CuSCN has received significant attention as an HTM in PSC due to the fact that it has a higher maximum hole mobility of about 0.1 cm^2^ V^−1^ s^−1^ which is far larger than of spiro-OMeTAD with a maximum hole mobility of 6 × 10^−5^ cm^2^ V^−1^ s^−1^. Moreover, CuSCN HTM has suitable energy levels (Figure 2) as well as affordable and simplified synthesis routes as compared to spiro-OMeTAD which makes it ideal for large-scale applications. In light of these observations, this review paper will focus on the recent progress of CuSCN HTM in PSCs.

## 3. Synthesis and Deposition of CuSCN HTM

**Figure 3:**
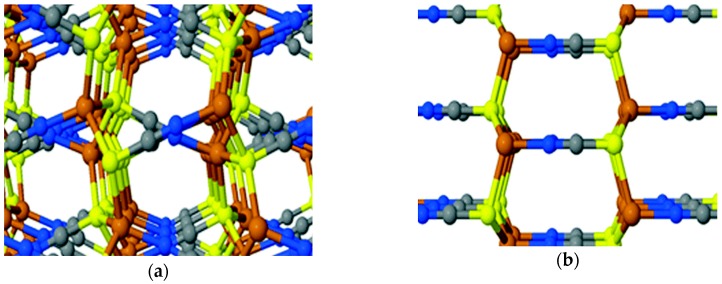
Bulk three-dimensional phases of CuSCN: (**a**) α-phase (orthorhombic); (**b**) β-phase (hexagonal) where brown sphere = Cu; yellow sphere = S; gray sphere = C; and blue sphere = N. Reproduced with permission from Reference [38]. Wiley 2017.

Copper thiocyanate is a p-type semiconductor with appealing characteristics such as a wide band gap of 3.6 V, high hole mobility of 0.01–0.1 cm^2^ V^−1^ s^−1^, high transparency, and high chemical stability [36]. CuSCN exists in α- and β-phase, with the structures given in Figure 3. It has been reported that β-phase is more stable and is easily accessible. The α- and β-phase can be classified as orthorhombic and hexagonal or rhombohedral structure, respectively [37]. Unlike other inorganic HTMs such as Cu_2_O, NiO and SnO, CuSCN is an efficient hole injection/extraction material as well as an electron blocking material due to its appropriate electronic levels. The excellent hole transporting properties of CuSCN are due to Cu 3d orbital with some hybridization from the S 3p orbitals near the valence band edge and the π^*^ antibonding orbital from the cyanide portion near the conduction band edge. Additionally, CuSCN can be modified easily due to its quasi-molecular property resulting in new materials with different properties [38]. This makes CuSCN a material of choice for various optoelectronic applications. 

CuSCN has been used in quantum dot-based light-emitting diodes [39], organic light-emitting diodes [40] and transparent thin-film transistors, and has yielded exceptional results [41]. Due to the simplified, affordable and straightforward techniques used to fabricate CuSCN HTMs, PSCs employing CuSCN HTMs have a greater opportunity to reach the commercial markets as compared to PSCs employing spiro-OMeTAD as HTM. 

**Figure 4:**
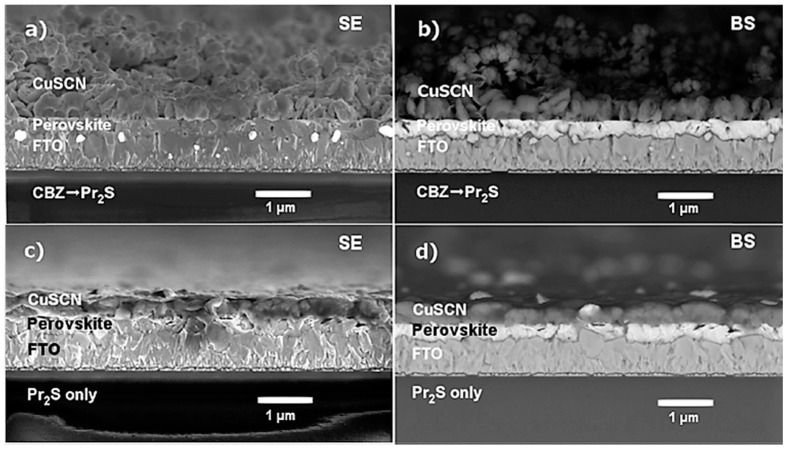
Scanning Electron Microscopy (SEM) cross-sectional images of FTO/c-TiO_2_/CH_3_NH_3_PbI_3_/CuSCN assemblies that were fabricated by doctor blading a solution of CuSCN in dipropyl sulfide (**a**,**b**) on a chlorobenzene pre-wetted perovskite layer (CBZ→Pr_2_S) and (**c**,**d**) on a dry perovskite layer (Pr_2_S only). SE: secondary electron images; BS: backscattered electron images. Reproduced with permission from Reference [49]. Elsevier 2017.

To that end, simple deposition techniques such as doctor blading, spin coating, spray coating, and electrodeposition have been used in depositing CuSCN HTMs [42,43,44] which augur well with the commercialization of these devices. However, it has been reported that solvent selection for solution-based deposition methods plays a crucial part in the overall photovoltaic performance of PSCs employing CuSCN HTMs. Polar solvents, such as ethyl sulfide, dipropyl sulfide, and diethyl sulfide [45,46,47] have been used in the deposition of CuSCN HTMs, with dipropyl sulfide being the most widely used. The use of a wide range of solvents is very limited in case of CuSCN as it is only soluble in polar solvents. However, these polar solvents tend to dissolve the perovskite layer as well, which results in very thin perovskite layers and, consequently, lowers the power conversion efficiency (PCE). Qin et al. [48] used doctor blading to deposit CuSCN using dipropyl sulfide solvent. A film thickness of 600 nm was reported and there was some evidence of dissolution of the perovskite on the CuSCN. A PCE of 12.4% was exhibited by the CuSCN-based PSC, with a V_OC_ of 1.016 V and short-circuit current density (J_SC_) of 19.7 mAcm^−2^. The number of deposition cycles did not have an effect on V_OC_ and J_SC,_ however, it led to an improved FF. In a move to reduce the dissolution of the perovskite layer in the HTM, Sepalage et al. [49] applied a chlorobenzene protective layer on the perovskite prior to deposition of CuSCN by doctor blading. The PSC exhibited a PCE of 9.6% which to the best of our knowledge is the highest achieved to date in a planar (n-i-p) CuSCN-based PSC device. The doctor blading method involved wetting the perovskite layer with chlorobenzene prior to depositing the CuSCN in dipropyl sulfide. This resulted in the formation of a thick uniform CuSCN film. Whilst the device without the chlorobenzene buffer resulted in relatively thin films as shown here in Figure 4. Figure 4, clearly shows that dissolution of the perovskite layer occurs when only dipropyl sulfide is used for deposition of CuSCN. 

**Figure 5:**
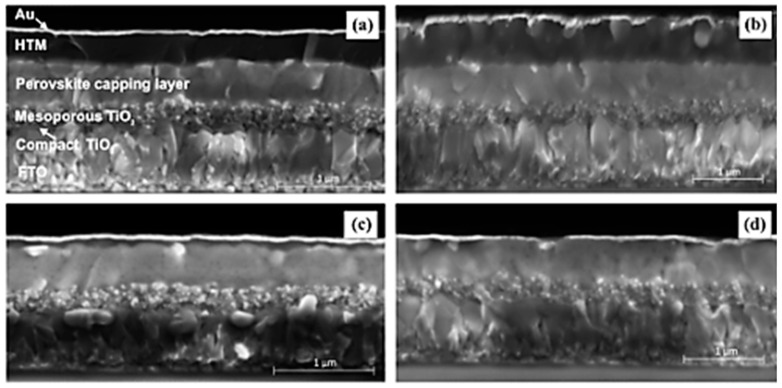
(**a**) SEM cross-section of spiro-OMeTAD-based HTM; (**b**) SEM cross-section of HTM deposited by doctor-blade technique; (**c**) SEM cross-section of HTM deposited by spin-coating technique; (**d**) SEM cross-section of PSC without HTM. Reproduced with permission from Reference [44]. American Chemical Society 2016.

Doctor blading is a simple, solution-based and low-cost thin film deposition method that is suitable for roll-to-roll (R2R) device fabrication. It is one of the common deposition methods used for depositing CuSCN HTMs. However, Yang et al. [42] reported that damage of the perovskite layer induced by doctor blading of CuSCN HTM in planar (n-i-p) PSC devices may be the reason for low performance of planar (n-i-p) devices as compared to inverted (p-i-n) devices. Yang and associates reported an economical simple spray deposition method employing a homemade spray equipment. The CuSCN was dissolved in dilute dipropyl sulfide. The deposition process involved spraying CuSCN at a distance of about 6 cm for 2 s and drying at 80 °C for 5 s. The thickness of the HTM layer was controlled by the number of deposition cycles. Spray deposition resulted in the formation of a uniform CuSCN layer with a thickness of about 50 nm. There was no significant damage to the perovskite layer which was attributed to the minimum contact of the perovskite layer to the dipropyl sulfide solvent. A PCE of 17% was reported for a Au/CuSCN/perovskite/TiO_2_/FTO device and a V_OC_ of about 1 V which is typical of organic HTM-based PSCs without additives. Spray coating is a simple deposition method which is compatible with large-scale fabrication. However, Madhavan et al. [44] proved CuSCN deposited by doctor blading can still attain high PCEs in mesoscopic (n-i-p)-based PSC devices. In their work, they reported a PCE of 16.6% for a doctor-bladed CuSCN-based PSCs while the spin-coated CuSCN-based device had a 15.4%. The difference in PCE was attributed to different HTM film thickness, with the doctor bladed CuSCN-based film having a thickness of about 500 nm while the spin-coated CuSCN-based had a thickness of about 30 nm as shown in Figure 5. The spin-coated CuSCN thin films were uniform and managed to effectively extract holes even though the films were limited to only 30 nm. When compared to doctor blading, spin coating is not cost effective and is not compatible with a large-scale manufacture where high-throughput fabrication is required. The non-cost effectiveness of spin coating can be attributed to the requirement of large volumes of solvents and raw materials used in spinning coating [50]. 

Another method which has been used for deposition of CuSCN HTM in PSCs is electrodeposition. Electrodeposition is a simple method which is low cost and is compatible with large-scale fabrication [51]. Shlenskaya et al. [52] reported an electrodeposited CuSCN blocking layer from a solution of CuSO_4_, Na_2_EDTA, and NaSCN in a three-electrode cell. The electrodeposition mechanism proceeded via nucleation and growth of the resulting CuSCN thin films. Formation of a porous CuSCN involved spin coating the sacrificial polystyrene template on *b*-CuSCN/ITO substrates from a water/ethanol suspension in the presence of a surfactant. This was followed by electrodeposition of CuSCN into the template at −0.1 V and with the thickness of the deposited film dependent on the potentiostatic deposition time. They obtained a CuSCN film thickness which ranged from 400 to 1400 nm for 0.5–3 layers of CuSCN. Porous CuSCN can be applied in mesoscopic (p-i-n) PSC devices. Nevertheless, Shlenskaya and associates did not report on the optical, stability properties as well as the photovoltaic performance of porous CuSCN HTM-based PSCs. Hence, there is a need to conduct more research to evaluate the optical stability properties and photovoltaic performance of electrodeposited porous CuSCN-based PSC. 

As we have mentioned earlier, finding an ideal solvent for deposition CuSCN HTMs has been a challenge. Research efforts have been dedicated to finding an ideal solvent which can dissolve CuSCN without damaging the perovskite layer. Recently, Murugadoss et al. [53] investigated the suitability of various solvent mixtures in dissolving CuSCN prior to deposition. The solvents were mixed in the following ratios; dipropyl sulfide and chlorobenzene (1:1); isopropanol and methylammonium iodide (MAI) (10 mg/mL); dipropyl sulfide, isopropanol and MAI ((1:2) +10 mg/mL) which were assigned S2, S3 and S4, respectively. The pristine dipropyl sulfide solvent was assigned S1. The photovoltaic parameters of the PSCs fabricated with different solvent ratios are shown here in Table 1. 

**Figure 6:**
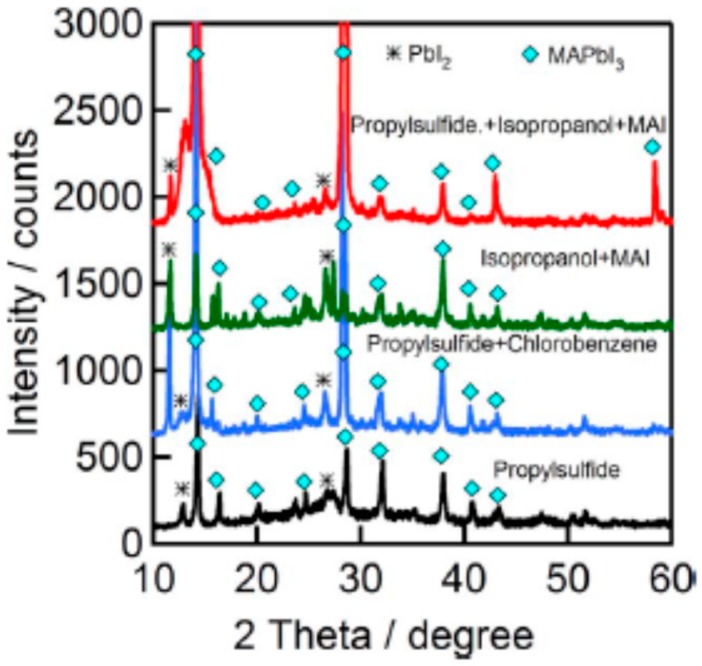
XRD patterns of FTO/bl-TiO_2_/m-TiO_2_/CH3NH3PbI3/CuSCN film, CuSCN was prepared using different solvents, S1, S2, S3 and S4, deposited by doctor blading method under 80 °C at air atmosphere. The peaks corresponding PbI2 and MAPbI3 are labelled by asterisk the (*) and (◊), respectively. Reproduced with permission from Reference [53]. Elsevier 2017.

Murugadoss et al. [53] found that the XRD peak due to PbI_2_ was of low intensity for S1 and higher for other solvents, as illustrated in Figure 6. Figure 6 shows PbI_2_ peaks of higher intensities for S2 and S3. This signified the high dissolution/decomposition rates of the perovskite structure in the presence of other solvents. Hence, the resulting efficiency of the fabricated PSCs was lower as shown in Table 1. Table 2 gives a summary of different solvents, methods and fabrication conditions used in the fabrication of CuSCN thin films. 

## 4. Architectures Used for CuSCN-Based PSCs

Device structure also plays an important role in the stability and efficiency of PSCs. The mesoscopic device architecture commonly demonstrates higher PCE as compared to planar device architecture. This can be attributed to the greater contact area with perovskite as well as decreased carrier transport distance in mesoscopic PSCs [62]. Having dipropyl sulfide as the solvent of choice during deposition of CuSCN limits the PSC device architecture which can be used. This results in inverted device architecture being the device structure of choice when it comes to CuSCN-based PSCs. However, to the best of our knowledge, there are no reports on mesoscopic (p-i-n) CuSCN-based PSCs. CuSCN films were first fabricated on the mesoporous structure by Ito et al. [63] and the structure proposed was FTO/compact TiO_2_/mesoporous TiO_2_/CH_3_NH_3_PbI_3_/CuSCN/Au which achieved a low PCE of 4.85%. Even though mesoscopic structure-based PSCs with spiro-OMeTAD HTM have exhibited high efficiency of 22.1%, they require high temperature during fabrication of mesoporous TiO_2_ layer which is not good for flexible PSCs [64] and does not bode well with characteristics of an ideal HTM. Hence, regular and inverted planar architectures were taken into consideration.

The first inverted planar architecture dates back to 2013; it was reported by Chen et al. [27] where it was applied in PSCs using PEDOT:PSS as the HTM. Unfortunately, their device degraded abruptly due to the acidic and hygroscopic nature of PEDOT:PSS. The inverted planar architecture was also introduced in inorganic HTM-based PSCs and has shown promising results as it requires low-temperature processing conditions. This makes it compatible with flexible substrates and moreover, inverted planar devices exhibit high FF [65,66]. Inverted planar architecture offers an easy device structure which is compatible with large-scale fabrication. Furthermore, it has comparable conversion efficiency with a mesoporous device structure, and negligible hysteresis effect made this structure the best so far [57,58]. The regular planar-based CuSCN PSCs, on the one hand, have exhibited high conversion efficiency and stability. Additionally, the planar structure is characterized by low-temperature processing requirements, low-cost, tunable device performance and an architecture that is capable of employing different interfacial materials [67,68]. In the following sub-sections, we discuss some of the regular planar and inverted planar-based CuSCN films structures and their device performances that have been reported to date.

### 4.1. n-i-p Architecture of CuSCN-Based PSCs

In 2014, Chavhan et al. [69] worked on organo-metal halide PSCs with CuSCN as the inorganic hole selective content and glass/FTO/TiO_2_/CH_3_NH_3_PbI_3−x_Cl_x_/CuSCN/Au as the device architecture. The fabricated planar PSCs were annealed at different temperatures; thereafter, solar cell characterization was performed. In their work, they discovered that PSC device annealed at 110 °C showed improved performance with a PCE of 6.4%. The photovoltaic performance parameters of other PSCs with planar structure glass/FTO/TiO_2_/CH_3_NH_3_PbI_3−x_Cl_x_/CuSCN/Au annealed at different temperatures are shown here in Table 3. 

**Figure 7:**
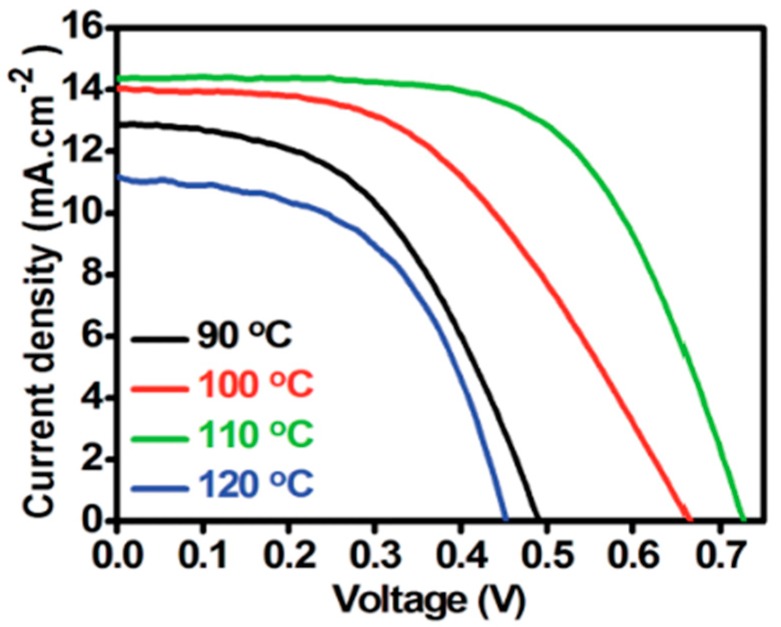
J-V curve of solar cells annealed at different temperatures under illumination of 100 mWcm^−2^ simulated sun irradiation (1.5 AM). Reproduced with permission from Reference [69]. Royal Society of Chemistry 2014.

It is clearly evident from Table 3 and Figure 7 that annealing had a great impact on solar cell performance as it has exhibited different photovoltaic performances at different annealing temperatures. It is also evident from Table 3 that the planar PSC structure yielded impressive results and even achieving a good FF of 61.7% which signifies good carrier selectivity. The efficiency of the CuSCN-based PSC was comparable to the first spiro-OMeTAD-based PSC with an efficiency of 9.7% [8]. However, the Voc of the planar PSC structures was a major setback, with a reported low value of 0.45 V which is indicative of short diffusion length of <0.75 V for CuSCN. The low Voc was attributed to high recombination rates originating from both perovskite material and CuSCN inorganic HTM. It is highly recommended to ensure that the charge carrier extraction in selective contacts is well-balanced in order to enhance the performance of planar heterojunction PSC device to reach high photocurrents. 

Arora et al. [70] reported a CuSCN-based PSC which exhibited high efficiency of 20.3% and this was attributed to reduced contact time between the perovskite layer and solvent during HTM deposition. The reduced the interaction between the perovskite material and the solvent used for dissolution of CuSCN was made possible by using a dynamic deposition approach whereby, the solvent is evaporated rapidly as compared to the conventional deposition approach. They reported that the rapid evaporation of solvent induced nucleation and growth of CuSCN nanostructures and high density of nucleation formed compact and thin CuSCN films with nano-crystallites. This deposition approach has potential to improve the stability, as well as the photovoltaic performance of inorganic HTM-based PSCs since perovskite dissolution in solvents used in HTM deposition has been one of their major drawbacks.

**Figure 8:**
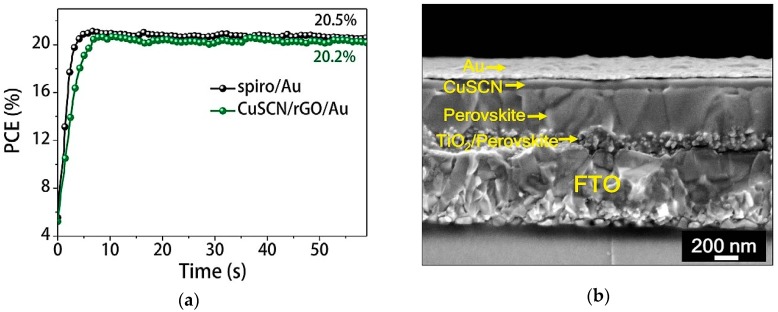
(**a**) The maximum power point (MMP) tracking for 60 s yielded a stabilized efficiency of 20.5 and 20.3% for spiro-OMeTAD and CuSCN-based PSCs; (**b**) SEM cross-sectional micrograph of CuSCN HTM-based PSC. Reproduced with permission from Reference [70]. AAAS 2017.

Arora and associates also gave an insight on issues of instability in CuSCN-based PSCs. In their work, they introduced a thin spacer layer of reduced graphene oxide (rGO) using spin coating before depositing the Au layer with a resulting overall structure: FTO/compact TiO_2_/mesoporous TiO_2_/C_s_FAMAPbI_3−x_Br_x_/CuSCN/rGO/Au, the SEM cross-sectional micrograph is shown in Figure 8. Figure 8 also shows the maximum power point tracking for 60 s which yielded a stabilized efficiency of spiro-OMeTAD and CuSCN PSCs devices. 

Thin spacer layer was introduced after realizing that the degradation was not caused by the solvent used for dissolving the CuSCN but rather the oxidation of the gold cathode when the CuSCN-based device is undergoing the light soaking test. In previous reports, it was reported that the solvent used in deposition of CuSCN layer tend to dissolve the perovskite material but the X-ray photoelectron spectroscopy (XPS) reported by Arora and co-workers proved the results otherwise. The approach of introducing a thin spacer layer improved the carrier extraction and collection processes which resulted in a PCE of 20.4% with Jsc of 23.40 mAcm^−2^, Voc of 1.10 V and FF of 77.2%. Baranwal et al. [71] replaced the Au electrode with a low temperature-processed carbon electrode in a mesoscopic CuSCN-based PSC for the first time. CuSCN reduces the difference in energy between the valence band of the CH_3_NH_3_PbI_3_ perovskite and the Fermi level of the carbon electrode which allows smooth path for transport of holes. The use of CuSCN resulted in reduced recombination losses and this led to a moderate efficiency of 12.4%. However, when compared to CuSCN-based PSC reported by Arora et al. [70], CuSCN-based PSC with carbon electrode lags far behind in terms of efficiency. 

### 4.2. Inverted (p-i-n) Architecture of CuSCN-Based PSCs

**Figure 9:**
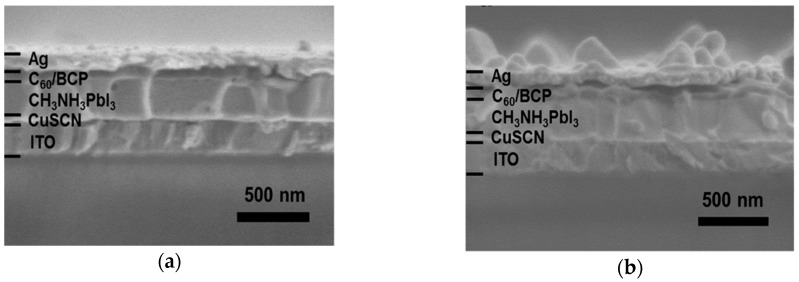
(**a**) SEM cross-sectional image of a device fabricated with one-step fast deposition technique; (**b**) SEM cross-sectional image of a device fabricated with the two-step technique deposition technique. Reproduced with permission from Reference [72]. American Chemical Society 2015.

In 2015, Ye et al. [72] reported on CuSCN-based inverted planar perovskite solar cell. Deposition of CuSCN was carried via a one-step deposition for device A, while two-step sequential deposition process was used for device B. Figure 9 shows the SEM cross-sectional images of the two devices and Table 4 presents the respective photovoltaic performance parameters. Device B was found to degrade rapidly due to percolation of air and moisture to the perovskite layer.

One of the inherent advantages of organic-based HTMs is their low-temperature processability. This has been a challenge in inorganic HTMs as they require high temperatures for deposition. Recently, Xiong et al [46] reported an inverted PSC which employed CuSCN modified PEDOT:PSS as HTM resulting in an HTM bilayer. Their approach was similar to that reported by Hu et al. [73], where PEDOT:PSS and CuI were used as bilayer HTM which resulted in improved stability and efficiency. Xiong et al. [46] reported a PCE of 10.09%, which was relatively higher than of the PEDOT:PSS-based PSC control (9.1%). The CuSCN modified PEDOT:PSS-based device exhibited improved V_OC_ and this could be attributed to a better-matched energy alignment between MAPbI_3_ and CuSCN modified PEDOT:PSS HTM. The PEDOT:PSS and CuSCN were spin-coated and annealed at 120 °C and 60 °C respectively. When compared to annealing temperatures mentioned in Table 3, the CuSCN modified PEDOT:PSS-based performed better at relatively similar annealing temperatures. Wang et al. [74] also reported improved photovoltaic performance for a CuSCN/CuI composite-based PSC. The CuSCN/CuI composite-based PSC had an efficiency of 18.76% as compared to 14.53% and 16.66% for CuI-based PSC and CuSCN-based PSC respectively. Presence of CuSCN resulted in formation of good quality HTM thin films which resulted in improved efficiency. Modification of CuSCN HTM might be one avenue that can be explored in order to have improved HTMs which meet properties of ideal HTMs, as well as being cost-effective. Table 5 shows the summary of some CuSCN-based PSCs and their photovoltaic performances. 

Table 5 shows the CuSCN HTM-based PSCs with their different device gestalts, the photovoltaic performances and the year of publication. The device with the lowest efficiency is 4.85% and was reported in 2014 and the device with the highest efficiency was reported in 2018 with an efficiency of 20.39%. This shows the conversion efficiency of CuSCN HTM has rapidly improved in the past 5 years using different synthesis methods and device architectures. Additionally, Table 6 presents the comparative PSC device performance employing different HTMs.

It is clearly evident from Table 6 that PSCs employing PTAA and spiro-OMeTAD HTMs have achieved the maximum PCE of 22.1% and 21.6%, respectively, whilst the PSC employing CuSCN HTM has achieved a noble efficiency of 20.4% in a mesoscopic device architecture which is the most promising of the fabricated CuSCN HTM. Though the efficiency of PSC employing inorganic HTM such as CuSCN is still lower as compared to the spiro-OMeTAD and PTAA HTM, inorganic HTMs are more economical. Spiro-OMeTAD and PTAA require additives, moreover these HTMs are not compatible with large-scale manufacturing processes. Yet the preparation of PSC employing inorganic HTMs is far much economical and is more feasible for solar panel industry. It would be ideal to fabricate PSC consisting of HTM bilayers or composite HTMs such as CuSCN/CuI and CuSCN/NiO.

## 5. Stability of CuSCN-Based PSCs

Stability issue is one of the major drawbacks that limit the perovskite-based solar cells from commercialization. Besides perovskite materials, HTMs are also known to contribute to the long-term stability of PSCs. For example, the use of additives in spiro-OMeTAD results in reduced stability of PSCs. This is due to the hygroscopic nature of the additives which leads to degradation of the HTM as well as the perovskite layer when exposed to moisture. The morphology of spiro-OMeTAD is also a cause of concern as it has pin-holes which are a source of degradation in PSCs [50,81]. PEDOT:PSS is the choice HTM for inverted (p-i-n) PSC devices. However, it is a source of instability in PSCs as it is hygroscopic and acidic in nature [82,83]. Thermal stability is also of paramount importance in HTMs since solar panels are exposed to very high temperatures when operating. Therefore, solar panels must be able to withstand a temperature of up to 85 °C according to international standards [84]. Introduction of inorganic HTMs has not only been a move to lower cost of PSCs but as a way to improve the stability of PSCs as well. Recently, Yang et al. [42] reported a remarkable stability of a CuSCN-based PSC without encapsulation. The PCE was reduced by 5.8% only when the CuSCN-based PSC was exposed to 30% relative humidity for 100 days indoors. When compared to the spiro-OMeTAD-based control PSC which reduced by 30%, it is clear that the CuSCN-based performed exceptionally well. In terms of thermal stability, the CuSCN-based PSC also fared much better than the spiro-OMeTAD-based control PSC. This is evident by the reduction of PCE from 16.46% to 14.32% for CuSCN-based PSC while the spiro-OMeTAD reduced to as low as 5.29% from 17.63% after encapsulation at 120 °C for 5 min. 

**Figure 10:**
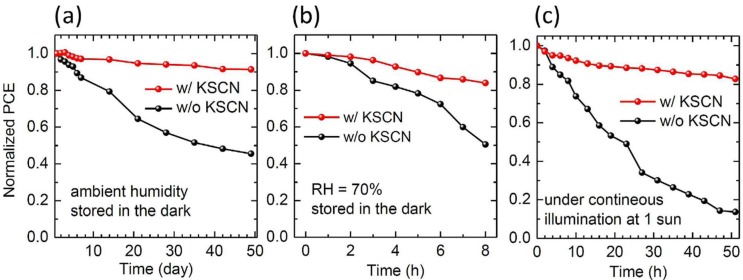
Time-dependent PCE of PSCs depending on post-treatment with KSCN at different condition of (**a**) ambient atmosphere with low humidity (the devices kept in the vacuum chamber in the dark before and after measurements), (**b**) relative humidity (RH) of 70% and (**c**) continuous illumination under one sun (the devices were encapsulated). Reproduced with permission from Reference [76]. Elsevier 2019.

Lyu et al. [76] reported better stability of KSCN post-treated CuSCN-based inverted planar PSC. The KSCN post-treated CuSCN PSC managed to retain about 90% of its initial PCE after 50 days in ambient humid conditions in the dark as compared to 60% for untreated CuSCN-based PSC as shown in Figure 10 a. The encapsulated KSCN post-treated CuSCN-based PSC also fared better than the untreated CuSCN-based PSC when exposed to continuous illumination under one sun as shown in Figure 10 b. The relative better stability of the KSCN post-treated CuSCN-based PSC was attributed to reduced trapped charges at the perovskite/CuSCN interface. Post-treating CuSCN film with KSCN did not only improved stability, it also improved photovoltaic performance of the cell. This might have been due to presence of excess SCN^−^ from the KSCN, since excess SCN^−^ increase the hole conductivity of CuSCN. This is evident from the reported 14.90% PCE for KSCN post-treated CuSCN-based PSC as compared to 11.90% for untreated CuSCN-based PSC.

**Figure 11:**
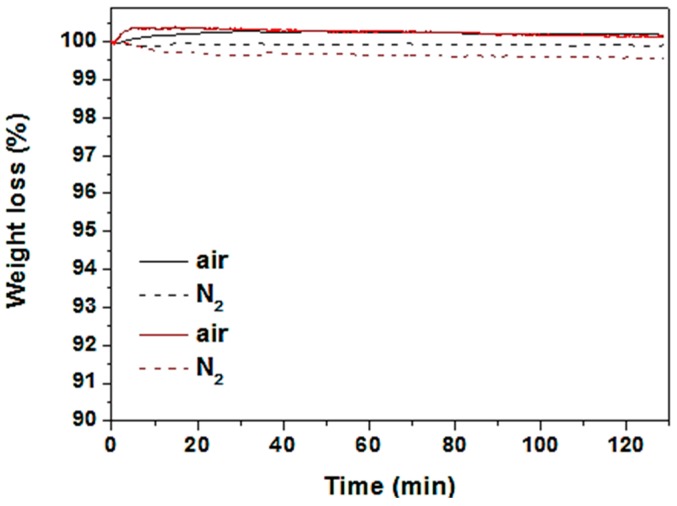
TGA curves of pristine FAPbI_3_ (black) and CuSCN (red) powders at 125 °C in air for 2 h, equivalent to the thermal stability testing conditions. Reproduced with permission from Reference [75]. Wiley 2016.

Jung et al. [75] tested the intrinsic thermal stability of CuSCN and spiro-OMeTAD materials using thermogravimetric analysis (TGA). There was relatively no observed weight loss for both samples exposed to air or nitrogen at 125 °C for 2 h as shown in Figure 11. They went on to investigate the thermal stability of CuSCN-based PSC at 125 °C for 120 min in air at 40% relative humidity in the dark. The CuSCN-based PSC managed to retain more than 60% of its initial efficiency while the spiro-OMeTAD-based control PSC had a dramatic efficiency reduction from 18.4% to 4.6%. 

This goes to show that when it comes to thermal stability, CuSCN HTM performs much better than spiro-OMeTAD. Recently, Chowdhury et al. [60] reported a modified CuSCN/rGO bilayer-based PSC. The rGO/CuSCN-based PSC exhibited better stability under light soaking test for 100 h. It had an efficiency reduction of about 10% as compared to the pristine CuSCN-based PSC which had about 50% efficiency reduction under the same conditions. Modification of HTMs might result in materials with improved properties. In general, CuSCN-based PSCs exhibit better stability as compared to organic-based PSCs. However, there is a need to test the stability of PSCs under the standard thermal test conditions rather than doing it under shorter periods of times. It would also result in clear comparisons among tested solar cells.

## 6. Recommendations and Conclusions 

Perovskite solar cells have shown promising characteristics as an alternative to expensive silicon solar technology with the high efficiency of 25.6%. However, the drawbacks caused by sensitivity of organic–inorganic halide-based PSCs to UV radiation and moisture make these solar cells inapplicable for large-scale practical applications. Research on developing inexpensive and stable materials for hole transport materials, such as inorganic semiconductors to replace organic HTMs like spiro-OMeTAD, has been the main priority. Research and development of inorganic HTMs have received significant attention due to the fact that inorganic HTMs are more stable, affordable, have high hole mobility, wide band gap, high transparency, high chemical stability, can be deposited at low temperatures and have simple synthetic routes which can be tailored to industrial scale. In lieu of these observations, this review paper has provided the current progress on the development of PSCs employing inorganic HTM. More specifically, the current progress on PSC employing CuSCN HTM has been provided in detail. This review paper has shown that CuSCN HTM thin films can be deposited by simple and affordable techniques. In this review, we have shown that CuSCN HTM thin films have been deposited by doctor blading, spin coating, spray deposition, and even electrodeposition. Our review has shown that the highest PSC PCE was achieved with a device that had thin films deposited by spray deposition (17.10%), followed by doctor blading (16.6%) and spin coating (15.6%). We also, discovered that the nature of the solvent used in the deposition of these thin films had an impact on the photovoltaic performance of the PSC. To that end, various solvents have been reported to try to reduce the dissolution rates of the perovskite material. Various solvents like dipropyl sulfide, chlorobenzene, isopropanol, methylammonium iodide (MAI), DMSO, and deionized water have been explored to date. Additionally, various solvent mixtures have also been explored with the highest PCE in PSC being reported for PSC with of 10.07% achieved with isopropanol and MAI. However, the search for an ideal solvent to deposit CuSCN thin films is still on. In this review, we have also, shown that device architectures in PSCs employing CuSCN as HTM also play a crucial role in the photovoltaic performance of the PSC. Our review has shown that the mesoscopic device architecture commonly employed for PSCs with spiro-OMeTAD which has a PCE of 21.1% whilst the PSC employing CuSCN as HTM has a reported PCE of 4.85% and a high PCE of 20.3% was reported for n-i-p architecture. We also reported planar and inverted planar device architectures employing CuSCN as HTM; the highest conversion efficiency reported for planar device architecture is 9.6%, whilst that of an inverted planar device is 15.6%. This shows that mesoporous device architecture is by far the best architecture to use for CuSCN-based PSCs as all the top five devices reported with the highest efficiency are of the mesoscopic devices. 

It has been reported that inorganic HTMs are more thermally stable when exposed to weather elements as compared to organic HTMs such as spiro-OMeTAD and PEDOT:PSS. CuSCN being an inorganic HTM is no exception. In this review, CuSCN has shown remarkable stability when exposed to weather elements. However, it is difficult to give a comparative analysis of the stability of CuSCN-based PSCs, and this is due to the reported stability tests being carried out at different test conditions. Perhaps carrying out stability tests under standard test conditions might give a more accurate comparison. This paper has provided a detailed review of the progress achieved to date on the use of CuSCN as HTM in PSCs. The triumphs and challenges of the PSC employing CuSCN as HTM has been discussed here in detail. 

## Figures and Tables

**Table 1:** The photovoltaic parameters of the PSCs fabricated with different solvent ration.

Solvent Mixture	Sample Codes	J_SC_ (mAcm^−2^)	V_OC_ (V)	FF (%)	PCE (%)
**pristine dipropyl sulfide**	S1	18.76	0.92	56.0	9.79
**dipropyl sulfide and chlorobenzene (1:1)**	S2	18.93	0.95	49.0	8.97
**isopropanol and methylammonium iodide (MAI) (10 mg/mL)**	S3	18.31	0.84	55.0	8.53
**isopropanol and MAI ((1:2) +10 mg/mL)**	S4	19.42	0.92	56.0	10.07

FF = Fill factor.

**Table 2:** Fabrication of CuSCN films using different starting material and solvents.

Starting Material	Solvent Used	Duration	Temp	Additives	Deposition	Thickness (nm)	Ref.
**CuSO_4_, KSCN**	DI water		RT	EDTA	Electrochemical	70–90	[54]
**CuSO_4_,** **KSCN**	DI water		RT	DEA	Electrochemical		[55]
**CuSO_4_,** **KSCN**	DI water		RT	TEA, EDTA, CDTA, NTA	Electrochemical	80	[56]
**CuSCN**	Dipropyl sulfide	4 h	RT		Spin-coating	300	[57]
**CuSCN**	Dipropyl sulfide	Overnight	RT		Doctor-blading	~400	[58]
**CuSCN**	Dipropyl sulfide	5 h	RT		Spin-coating	13	[59]
**CuSCN**	Dipropyl sulfide	Overnight	RT		Spin-coating Doctor-blading	~30 ~500	[44]
**CuSCN**	DMSO	2 h	RT		Spin-coating		[47]
**CuSCN**	Dipropyl sulfide; Dipropyl sulfide + Chlorobenzene; Isopropanol + MAI; Dipropyl sulfide + isopropanol + MAI	Overnight	RT		Doctor-blading	450	[53]
**CuSCN**	Diethyl sulfide		RT		Spin-coating	10–40	[60]
**CuSCN**	Diethyl sulfide, Ammonia	1 h	50 °C		Spin-coating	3–5	[61]

RT = room temperature; Temp = temperature; DI = deionized DMSO = dimethyl sulfoxide.

**Table 3:** Photovoltaic performance parameters of glass/FTO/TiO_2_/CH_3_NH_3_PbI_3−X_/CuSCN/Au device annealed at different temperatures.

Annealing Temp. (°C)	Jsc (mAcm^−2^)	Voc (V)	FF (%)	PCE (%)
**90**	13.04	0.49	49.0	3.1
**100**	14.27	0.67	48.1	4.5
**110**	14.4	0.73	61.7	6.4
**120**	11.1	0.45	53.8	2.7

Temp. = temperature.

**Table 4:** The photovoltaic performance of Device A and B deposited using different techniques.

Device	Jsc (mAcm^−2^)	Voc (V)	FF (%)	PCE (%)
**Device A**	21.9	1.00	75.8	16.6
**Device B**	21.4	0.92	68.1	13.4

**Table 5:** Summary of photovoltaic device performance of CuSCN HTMs for PSCs.

Device Architecture	Device Type	Jsc (mA/cm^2^)	Voc (V)	FF (%)	PCE (%)	Year	Reference
**FTO/compact TiO_2_/mesoporousTiO_2_/C_s_FAMAPbI_3−x_Br_x_/CuSCN/Al_2_O_3_/rGO/Au**	M	23.39	1.10	76.1	20.39	2017	[70]
**FTO/blocking TiO_2_/mesoporous TiO_2_/(FAPbI_3_)_0.85_(MAPbBr_3_)_0.15_/CuSCN/Au**	M	23.1	1.04	75.3	18.0	2016	[75]
**FTO/compact TiO_2_/mesoporousTiO_2_/CH_3_NH_3_PbI_3_/CuSCN/Au**	M	23.10	1.01	73.1	17.10	2017	[42]
**ITO/CuSCN/MAPbI_3−x_Cl_x_/PC_61_BM/PEI/Ag**	IP	20.76	1.10	73.0	16.66	2018	[74]
**ITO/CuSCN/CuI/MAPbI_3−x_Cl_x_/PC_61_BM/PEI/Ag**	IP	22.33	1.11	76.0	18.76	2018	[74]
**FTO/compact TiO_2_/mesoporousTiO_2_/(FAPbI_3_)_0.85_(MAPbBr_3_)_0.15_/CuSCN/Au**	M	21.80	1.10	69.2	16.6	2016	[44]
**ITO/CuSCN/CH_3_NH_3_PbI_3_/C60/BCP/Ag**	IP	21.9	1.00	75.8	15.6	2015	[72]
**ITO/CuSCN/MAPbI_3_/PCBM/BCP/Ag**	IP	19.20	1.01	77.0	14.90	2019	[76]
**ITO/rGO/CuSCN/CH_3_NH_3_PbI_3_/PCBM/BCP/Ag**	IP	18.21	1.03	76.1	14.28	2018	[60]
**FTO/dense-TiO_2_/mesoporous TiO_2_/MAPbI_3_/CuSCN/Carbon**	M	18.90	0.96	68.0	12.41	2018	[71]
**F:SnO_2_/TiO_2_/CH_3_NH_3_PbI_3_/CuSCN/Au**	M	19.7	1.02	62.0	12.4	2014	[48]
**ITO/CuSCN/CH_3_NH_3_PbI_3_/LiF/Ag**	IP	15.76	1.06	63.2	10.8	2015	[59]
**FTO/CuSCN-PEDOT:PSS/CH_3_NH_3_PbI_3_/Ag**	IP	17.6	0.86	71.7	10.09	2018	[46]
**FTO/blocking** **TiO_2_/mesoporous TiO_2_/MAPbI_3_/CuSCN/Au**	M	16.82	0.89	61.4	9.20	2018	[58]
**FTO/blocking TiO_2_/mesoporous TiO_2_/CH_3_NH_3_PbI_3_/CuSCN**	M	19.15	0.93	56.0	10.04	2017	[53]
**FTO/compact TiO_2_/CH_3_NH_3_PbI_3_/CuSCN/Graphite**	P	19.3	0.84	59.6	9.6	2017	[49]
**FTO/TiO_2_/CH_3_NH_3_PbI_3−x_Cl_x_/CuSCN/Au**	P	18.53	0.73	61.7	6.4	2014	[69]
**FTO/dense-TiO_2_/nanocrystalline** **TiO_2_/CH_3_NH_3_PbI_3_/CuSCN/u**	P	14.5	0.63	53.0	4.85	2014	[63]

M = Mesoscopic (n-i-p) device; P = Planar (n-i-p) device; IP = Inverted planar (p-i-n) device.

**Table 6:** Comparison of efficiency of CuSCN-based PSC with other HTMs.

HTM	Jsc (mAcm^−2^)	Voc (V)	FF (%)	PCE (%)	Reference
**CuSCN**	23.39	1.1	76.1	20.4	[70]
**Spiro-OMeTAD**	24.57	1.11	79,2	21.6	[2]
**PTAA**	24.1	1.1	81.90	22.1	[77]
**NiO**	20.2	1.06	81.3	17.3	[78]
**CuI**	22.8	1.01	73	16.8	[20]
**CuPc**	23.19	1.13	73.1	18.68	[79]
**Li_0.05_Mg_0.15_Ni_0.8_O**	22.68	1.12	77	19.58	[80]

## References

[1] Zhang L., Liu X., Li J., McKechnie S. (2018). Interactions between molecules and perovskites in halide perovskite solar cells. Sol. Energy Mater. Sol. Cells.

[2] Yang D., Yang R., Wang K., Wu C., Zhu X., Feng J., Ren X., Fang G., Priya S., Liu S.F. (2018). High efficiency planar-type perovskite solar cells with negligible hysteresis using EDTA-complexed SnO2. Nat. Commun..

[3] Ibn-Mohammed T., Koh S.C.L., Reaney I.M., Acquaye A., Schileo G., Mustapha K.B., Greenough R. (2017). Perovskite solar cells: An integrated hybrid lifecycle assessment and review in comparison with other photovoltaic technologies. Renew. Sustain. Energy Rev..

[4] Green M.A., Ho-Baillie A., Snaith H.J. (2014). The emergence of perovskite solar cells. Nat. Photonics.

[5] Chen Q., De Marco N., Yang Y., Song T.-B., Chen C.C., Zhao H., Hong Z., Zhou H., Yang Y. (2015). Under the spotlight: The organic-inorganic hybrid halide perovskite for optoelectronic applications. Nano Today.

[6] Da Y., Xuan Y., Li Q. (2017). Quantifying energy losses in planar perovskite solar cells. Sol. Energy Mater. Sol. Cells.

[7] Salhi B., Wudil Y.S., Hossain M.K., Al-Ahmed A., Al-Sulaiman F.A. (2018). Review of recent developments and persistent challenges in stability of perovskite solar cells. Renew. Sustain. Energy Rev..

[8] Kim H.S., Lee C.R., Im J.H., Lee K.B., Moehl T., Marchioro A., Moon S.J., Humphry-Baker R., Yum J.H., Moser J.E. (2012). Lead iodide perovskite sensitized all-solid-state submicron thin film mesoscopic solar cell with efficiency exceeding 9%. Sci. Rep..

[9] Kojima A., Teshima K., Shirai Y., Miyasaka T. (2009). Cells, Organometal Halide Perovskites as Visible-Light Sensitizers for Photovoltaic. J. Am. Chem. Soc..

[10] Sun X., Zhao D., Li Z. (2018). Recent advances in the design of dopant-free hole transporting materials for highly efficient perovskite solar cells. Chin. Chem. Lett..

[11] Hawash Z., Universitet K., Ono L.K., Qi Y. (2017). Recent Advances in Spiro-MeOTAD Hole Transport Material and Its Applications in Organic-Inorganic Halide Perovskite Solar Cells. Adva.

[12] Galatopoulos F., Savva A., Papadas I.T., Choulis S.A. (2017). The effect of hole transporting layer in charge accumulation properties of p-i-n perovskite solar cells. APL Mater..

[13] Uribe J., Ramirez D., Osorio-Guillen J., Osorio J., Jaramilo F. (2016). CH3NH3CaI3 Perovskite: Synthesis, Characterization, and First-Principles Studies. J. Phys. Chem..

[14] Zhao X., Wang M. (2018). Organic hole-transporting materials for efficient perovskite solar cells. Mater. Today Energy.

[15] Yang X., Wang H., Cai B., Yu Z., Sun L. (2018). Progress in hole-transporting materials for perovskite solar cells. J. Energy Chem..

[16] Chen W.-Y., Deng L.-L., Dai S.-M., Wang X., Tian C.-B., Zhan X.-X., Xie S.-Y., Huang R.-B., Zheng L.-S. (2015). Low-cost solution-processed copper iodide as an alternative to PEDOT:PSS hole transport layer for efficient and stable inverted planar heterojunction perovskite solar cells. J. Mater. Chem. A.

[17] Sun J., Lu J., Li B., Jiang L., Chesman A.S.R., Scully A.D., Gengenbach T.R., Cheng Y.B., Jasieniak J.J. (2018). Inverted perovskite solar cells with high fill-factors featuring chemical bath deposited mesoporous NiO hole transporting layers. Nano Energy.

[18] Yang Y., Pham N.D., Yao D., Zhu H., Yarlagadda P., Wang H. (2018). Inorganic p-type semiconductors and carbon materials based hole transport materials for perovskite solar cells. Chin. Chem. Lett..

[19] Christians J.A., Fung R.C.M., Kamat P.V. (2014). An Inorganic Hole Conductor for Organo-Lead Halide Perovskite Solar Cells. Improved Hole Conductivity with Copper Iodide. J. Am. Chem. Soc..

[20] Sun W., Ye S., Rao H., Li Y., Liu Z., Xiao L., Chen Z., Bian Z., Huang C. (2016). Room-Temperature and Solution-Processed Copper Iodide as Hole Transport Layer for Inverted Planar Perovskite Solar Cells. Nanoscale.

[21] Li M.H., Yum J.H., Moon S.J., Chen P. (2016). Inorganic p-type semiconductors: Their applications and progress in dye-sensitized solar cells and perovskite solar cells. Energies.

[22] Han G., Du W.H., An B.L., Bruno A., Leow S.W., Soci C., Zhang S., Mhaisalkar S.G., Mathews N. (2018). Nitrogen doped cuprous oxide as low cost hole-transporting material for perovskite solar cells. Scr. Mater..

[23] Chen J., Park N.-G. (2018). Inorganic Hole Transporting Materials for Stable and High Efficiency Perovskite Solar Cells. J. Phys. Chem. C.

[24] Jaffe J.E., Kaspar T.C., Droubay T.C., Varga T., Bowden M.E., Exarhos G.J. (2010). Electronic and Defect Structures of CuSCN. J. Phys. Chem. C.

[25] Cho A., Park N. (2017). Impact of Interfacial Layers in Perovskite Solar Cells. ChemSusChem.

[26] Abrusci A., Stranks S.D., Docampo P., Yip H., Jen A.K., Snaith H.J. (2013). High-Performance Perovskite-Polymer Hybrid Solar Cells via Electronic Coupling with Fullerene Monolayers. Nano Lett..

[27] Jeng J., Chiang Y., Lee M., Peng S., Guo T., Chen P., Wen T. (2013). CH3NH3PbI3 Perovskite/Fullerene Planar-Heterojunction Hybrid Solar Cells. Adv. Mater..

[28] Wang Y.K., Jiang Z.Q., Liao L.S. (2016). New advances in small molecule hole-transporting materials for perovskite solar cells. Chin. Chem. Lett..

[29] Calió L., Kazim S., Grätzel M., Ahmad S. (2016). Hole-Transport Materials for Perovskite Solar Cells. Angew. Chem.-Int. Ed..

[30] Dhingra P., Singh P., Rana P.J.S., Garg A., Kar P. (2016). Hole-Transporting Materials for Perovskite-Sensitized Solar Cells. Energy Technol..

[31] Bui T., Ulfa M., Maschietto F., Ottochian A., Nghiêm M., Cio I., Goubard F., Pauporté T. (2018). Design of dendritic core carbazole-based hole transporting materials for e ffi cient and stable hybrid perovskite solar cells. Org. Electron..

[32] Vivo P., Salunke J.K., Priimagi A. (2017). Hole-Transporting Materials for Printable Perovskite Solar Cells. Materials.

[33] Jiang X., Yu Z., Zhang Y., Lai J., Li J., Gurzadyan G.G., Yang X., Sun L. (2017). High-Performance Regular Perovskite Solar Cells Employing Low-Cost Poly ( ethylenedioxythiophene ) as a Hole-Transporting Material. Sci. Total Environ..

[34] Mahmood K., Sarwar S., Mehran M.T. (2017). Current status of electron transport layers in perovskite solar cells: materials and properties. RSC Adv..

[35] Yu Z., Sun L. (2017). Inorganic Hole-Transporting Materials for Perovskite Solar Cells. Small Methods.

[36] Premalal E.V.A., Kannangara Y.Y., Ratnayake S.P., Nalin de Silva K.M. (2017). Facile Synthesis of Colored and Conducting CuSCN Composite Coated with CuS Nanoparticles. Nanoscale Res. Lett..

[37] Ezealigo B.N., Nwanya A.C., Simo A., Bucher R., Osuji R.U., Maaza M., Reddy M.V., Ezema F.I. (2017). A study on solution deposited CuSCN thin films: Structural, electrochemical, optical properties. Arab. J. Chem..

[38] Pattanasattayavong P., Promarak V., Anthopoulos T. (2017). Electronic Properties of Copper (I) Thiocyanate (CuSCN). Adv. Electron. Mater..

[39] Ding T., Wang N., Wang C., Wu X., Liu W., Zhang Q., Fan W., Sun X.W. (2017). Solution-processed inorganic copper(i) thiocyanate as a hole injection layer for high-performance quantum dot-based light-emitting diodes. RSC Adv..

[40] Zhang Q., Guo H., Feng Z., Lin L., Zhou J., Lin Z. (2010). Electrochimica Acta n-ZnO nanorods/p-CuSCN heterojunction light-emitting diodes fabricated by electrochemical method. Electrochim. Acta.

[41] Petti L., Pattanasattayavong P., Lin Y.H., Münzenrieder N., Cantarella G., Yaacobi-Gross N., Yan F., Tröster G., Anthopoulos T.D. (2017). Solution-processed p-type copper(I) thiocyanate (CuSCN) for low-voltage flexible thin-film transistors and integrated inverter circuits. Appl. Phys. Lett..

[42] Yang I.S., Sohn M.R., Sung S.D., Kim Y.J., Yoo Y.J., Kim J., Lee W.I. (2017). Formation of pristine CuSCN layer by spray deposition method for efficient perovskite solar cell with extended stability. Nano Energy.

[43] Bakr Z.H., Wali Q., Fakharuddin A., Schmidt-Mende L., Brown T.M., Jose R. (2017). Advances in hole transport materials engineering for stable and e ffi cient perovskite solar cells. Nano Energy.

[44] Madhavan V.E., Zimmermann I., Roldán-Carmona C., Grancini G., Buffiere M., Belaidi A., Nazeeruddin M.K. (2016). Copper Thiocyanate Inorganic Hole-Transporting Material for High-Efficiency Perovskite Solar Cells. ACS Energy Lett..

[45] Hatch S.M., Briscoe J., Dunn S. (2013). Improved CuSCN–ZnO diode performance with spray deposited CuSCN. Thin Solid Films.

[46] Xiong Q., Tian H., Zhang J., Han L., Lu C., Shen B., Zhang Y., Zheng Y., Lu C., Zeng Z. (2018). CuSCN modified PEDOT:PSS to improve the efficiency of low temperature processed perovskite solar cells. Org. Electron..

[47] Chaudhary N., Chaudhary R., Kesari J.P., Patra A. (2017). An eco-friendly and inexpensive solvent for solution processable CuSCN as a hole transporting layer in organic solar cells. Opt. Mater..

[48] Qin P., Tanaka S., Ito S., Tetreault N., Manabe K., Nishino H., Nazeeruddin M.K., Grätzel M. (2014). Inorganic hole conductor-based lead halide perovskite solar cells with 12.4% conversion efficiency. Nat. Commun..

[49] Sepalage G.A., Meyer S., Pascoe A.R., Scully A.D., Bach U., Cheng Y.B., Spiccia L. (2017). A facile deposition method for CuSCN: Exploring the influence of CuSCN on J-V hysteresis in planar perovskite solar cells. Nano Energy.

[50] Qiu L., Ono L.K., Qi Y. (2018). Advances and challenges to the commercialization of organic–inorganic halide perovskite solar cell technology. Mater. Today Energy.

[51] Ramírez D., Riveros G., Álvarez K., González B., Pereyra C.J., Dalchiele E.A., Marotti R.E., Ariosa D., Martín F., Ramos-barrado J.R. (2017). Electrochemical synthesis of CuSCN nanostructures, tuning the morphological and structural characteristics: From nanorods to nanostructured layers. Mater. Sci. Semicond. Process..

[52] Shlenskaya N.N., Tutantsev A.S., Belich N.A., Goodilin E.A., Grätzel M., Tarasov A.B. (2018). Electrodeposition of porous CuSCN layers as hole-conducting material for perovskite solar cells. Mendeleev Commun..

[53] Murugadoss G., Thangamuthu R., Senthil Kumar S.M. (2017). Fabrication of CH3NH3PbI3 perovskite-based solar cells: Developing various new solvents for CuSCN hole transport material. Sol. Energy Mater. Sol. Cells.

[54] Wang F., Chen D., Hu Z., Qin L., Sun X., Huang Y. (2017). In situ decoration of CuSCN nanorod arrays with carbon quantum dots for highly ef fi cient photoelectrochemical performance. Carbon.

[55] Wu W., Cui S., Yang C., Hu G., Wu H. (2009). Electrochemistry Communications Electrochemically superfilling of n-type ZnO nanorod arrays with p-type CuSCN semiconductor. Electrochem. Commun..

[56] Chappaz-Gillot C., Salazar R., Berson S., Ivanova V. (2013). Insights into CuSCN nanowire electrodeposition on flexible substrates. Electrochim. Acta.

[57] Lv Y., Guo Y., Zhang H., Zhou X., Chen H. (2018). Enhanced efficiency and stability of fully air-processed TiO_2_ nanorods array based perovskite solar cell using commercial available CuSCN and carbon. Sol. Energy.

[58] Karuppuchamy S., Murugadoss G., Ramachandran K., Saxena V., Thangamuthu R. (2018). Inorganic based hole transport materials for perovskite solar cells. J. Mater. Sci. Mater. Electron..

[59] Zhao K., Munir R., Yan B., Yang Y., Kim T., Amassian A. (2015). Solution-processed inorganic copper(i) thiocyanate (CuSCN) hole transporting layers for efficient p-i-n perovskite solar cells. J. Mater. Chem. A.

[60] Chowdhury T.H., Akhtaruzzaman M., Kayesh M.E., Kaneko R., Noda T., Lee J.J., Islam A. (2018). Low temperature processed inverted planar perovskite solar cells by r-GO/CuSCN hole-transport bilayer with improved stability. Sol. Energy.

[61] Wijeyasinghe N., Regoutz A., Eisner F., Du T., Tsetseris L., Lin Y.H., Faber H., Pattanasattayavong P., Li J., Yan F. (2017). Copper(I) Thiocyanate (CuSCN) Hole-Transport Layers Processed from Aqueous Precursor Solutions and Their Application in Thin-Film Transistors and Highly Efficient Organic and Organometal Halide Perovskite Solar Cells. Adv. Funct. Mater..

[62] Song Z., Phillips A.B., Heben M.J., Song Z., Watthage S.C., Phillips A.B., Heben M.J., Song Z., Watthage S.C., Phillips A.B. (2016). Pathways toward high-performance perovskite solar cells: Review of recent advances in organo-metal halide perovskites for photovoltaic applications. J. Photonics Energy.

[63] Ito S., Tanaka S., Vahlman H., Nishino H., Manabe K., Lund P. (2014). Carbon-Double-Bond-Free Printed Solar Cells from TiO_2_/CH3NH3PbI3/CuSCN/Au: Structural Control and Photoaging Effects. ChemPhysChem.

[64] Yao Y., Wang G., Liao L., Liu D., Zhou G., Xu C., Yang X., Wu R., Song Q. (2018). Enhancing the open circuit voltage of PEDOT:PSS-PC61BM based inverted planar mixed halide perovskite solar cells from 0.93 to 1.05 V by simply oxidizing PC61 BM. Org. Electron..

[65] Han J., Tu Y., Liu Z., Liu X., Ye H., Tang Z., Shi T., Liao G. (2018). Efficient and stable inverted planar perovskite solar cells using dopant-free CuPc as hole transport layer. Electrochim. Acta.

[66] Guo H., Huang X., Pu B., Yang J., Chen H., Zhou Y., Yang J., Li Y., Wang Z., Niu X. (2017). Efficiency enhancement in inverted planar perovskite solar cells by synergetic effect of sulfated graphene oxide (sGO) and PEDOT:PSS as hole transporting layer. RSC Adv..

[67] Castro E., Murillo J., Fernandez-delgado O., Echegoyen L. (2018). Progress in fullerene-based hybrid perovskite solar cells. J. Mater. Chem. C.

[68] Qin Q., Zhang Z., Cai Y., Zhou Y., Liu H., Lu X., Gao X., Shui L., Wu S., Liu J. (2018). Improving the performance of low-temperature planar perovskite solar cells by adding functional fullerene end-capped polyethylene glycol derivatives. J. Power Sources.

[69] Chavhan S., Miguel O., Grande H.J., Gonzalez-Pedro V., Sánchez R.S., Barea E.M., Mora-Seró I., Tena-Zaera R. (2014). Organo-metal halide perovskite-based solar cells with CuSCN as the inorganic hole selective contact. J. Mater. Chem. A.

[70] Arora N., Dar M.I., Hinderhofer A., Pellet N., Schreiber F., Zakeeruddin S.M., Grätzel M. (2017). Perovskite solar cells with CuSCN hole extraction layers yield stabilized efficiencies greater than 20%. Science.

[71] Baranwal A.K., Kanda H., Shibayama N., Ito S. (2018). Fabrication of fully non-vacuum processed perovskite solar cells using inorganic CuSCN hole-transporting material and carbon- back contact. Sustain. Energy Fuels.

[72] Ye S., Sun W., Li Y., Yan W., Peng H., Bian Z., Liu Z., Huang C. (2015). CuSCN-Based Inverted Planar Perovskite Solar Cell with an Average PCE of 15.6%. Nano Lett..

[73] Hu W., Dall’Agnese C., Wang X.-F., Chen G., Li M.-Z., Song J.-X., Wei Y.-J., Miyasaka T. (2018). Copper iodide-PEDOT:PSS double hole transport layers for improved efficiency and stability in perovskite solar cells. J. Photochem. Photobiol. A Chem..

[74] Wang H., Yu Z., Lai J., Song X., Yang X., Hagfeldt A., Sun L. (2018). One plus one greater than two: high-performance inverted planar perovskite solar cells based on a composite CuI/CuSCN hole-transporting layer. J. Mater. Chem. A.

[75] Jung M., Kim Y.C., Jeon N.J., Yang W.S., Seo J., Noh J.H., Il Seok S. (2016). Thermal Stability of CuSCN Hole Conductor-Based Perovskite Solar Cells. ChemSusChem.

[76] Lyu M., Chen J., Park N. (2019). Improvement of e ffi ciency and stability of CuSCN-based inverted perovskite solar cells by post-treatment with potassium thiocyanate. J. Solid State Chem..

[77] Yang W.S., Park B.W., Jung E.H., Jeon N.J., Kim Y.C., Lee D.U., Shin S.S., Seo J., Kim E.K., Noh J.H. (2017). Il Iodide management in formamidinium-lead-halide-based perovskite layers for efficient solar cells. Science.

[78] Park J.H., Seo J., Park S., Shin S.S., Kim Y.C., Jeon N.J., Shin H.W., Ahn T.K., Noh J.H., Yoon S.C. (2015). Il Efficient CH3NH3PbI3 Perovskite Solar Cells Employing Nanostructured p-Type NiO Electrode Formed by a Pulsed Laser Deposition. Adv. Mater..

[79] Duong T., Peng J., Walter D., Xiang J., Shen H., Chugh D., Mark N., Zhong D., Li J., Weber K.J. (2018). Perovskite Solar Cells Employing Copper Phthalocyanine Hole Transport Material with an Efficiency over 20% and Excellent Thermal Stability. ACS Energy Lett..

[80] Wu Y., Xie F., Chen H., Yang X., Su H., Cai M., Zhou Z., Noda T., Han L. (2017). Thermally Stable MAPbI_3_ Perovskite Solar Cells with Efficiency of 19.19% and Area over 1 cm^2^ achieved by Additive Engineering. Adv. Mater..

[81] Chen J., Cai X., Yang D., Song D., Wang J., Jiang J., Ma A., Lv S., Hu M.Z., Ni C. (2017). Recent progress in stabilizing hybrid perovskites for solar cell applications. J. Power Sources.

[82] Yu H., Wu Z., Huang X., Shi S., Li Y. (2018). Synergetic effects of acid treatment and localized surface plasmon resonance in PEDOT:PSS layers by doping HAuCl4 for efficient polymer solar cells. Org. Electron..

[83] Lee J.J., Lee S.H., Kim F.S., Choi H.H., Kim J.H. (2015). Simultaneous enhancement of the efficiency and stability of organic solar cells using PEDOT:PSS grafted with a PEGME buffer layer. Org. Electron..

[84] Meyer E., Mutukwa D., Zingwe N., Taziwa R. (2018). Lead-Free Halide Double Perovskites: A Review of the Structural, Optical, and Stability Properties asWell as Their Viability to Replace Lead Halide Perovskites. Metals.

